# Modeling of the Statistical Distribution of Tracheids in Conifer Rings: Finding Universal Criterion for Earlywood–Latewood Distinction

**DOI:** 10.3390/plants12193454

**Published:** 2023-09-30

**Authors:** Liliana V. Belokopytova, Dina F. Zhirnova, Bao Yang, Elena A. Babushkina, Eugene A. Vaganov

**Affiliations:** 1 Khakass Technical Institute, Siberian Federal University, 655017 Abakan, Russia; dina-zhirnova@mail.ru (D.F.Z.); babushkina70@mail.ru (E.A.B.); 2 Institute of Ecology and Geography, Siberian Federal University, 660036 Krasnoyarsk, Russia; eavaganov@hotmail.com; 3 School of Geographic and Oceanographic Science, Nanjing University, Nanjing 210093, China; yangbao@nju.edu.cn; 4 Department of Dendroecology, V.N. Sukachev Institute of Forest, Siberian Branch of the Russian Academy of Science, 660036 Krasnoyarsk, Russia

**Keywords:** quantitative woodanatomy, earlywood, latewood, transition wood, Mork criterion, cell wall thickness, cell radial diameter, generalized normal distribution

## Abstract

The quantitative description of growth rings is yet incomplete, including the functional division into earlywood and latewood. Methods developed to date, such as the Mork criterion for conifers, can be biased and arbitrary depending on species and growth conditions. We proposed the use of modeling of the statistical distribution of tracheids to determine a universal criterion applicable to all conifer species. Thisstudy was based on 50-year anatomical measurements of *Pinus sylvestris* L., *Pinus sibirica* Du Tour, and *Picea obovata* Ledeb. near the upper tree line in the Western Sayan Mountains (South Siberia). Statistical distributions of the cell wall thickness (CWT)-to-radial-diameter (D) ratio and its slope were investigated for raw and standardized data (divided by the mean). The bimodal distribution of the slope for standardized CWT and D was modeled with beta distributions for earlywood and latewood tracheids and a generalized normal distribution for transition wood to account for the gradual shift in cell traits. The modelcan describe with high accuracy the growth ring structure for species characterized by various proportions of latewood, histometric traits, and gradual or abrupt transition. The proportion of two (or three, including transition wood) zones in the modeled distribution is proposed as a desired criterion.

## 1. Introduction

In the concept of growth rings, which combines anatomical, periodic, causal, and evolutionary aspects, there are still many “white spots”, the interpretation and quantitative assessment of which are ambiguous [1,2,3,4]. Certain anatomical markers make it possible to recognize tree rings and judge causal relationships in their variability [3,5,6,7,8,9,10]. They arise due to the radial integrity of growth layers and/or the presence of earlywood and latewood zones [11,12,13], the transition between which is regulated by internal and external factors [8,14,15]. This zoning is functional, providing water conduction and mechanical strength, respectively [16,17,18]. Therefore, latewood content as the main carbon sink of the tree is important in modeling the global carbon cycle [14,19,20].

Several methods are used to quantify and separate growth ring zones, from the simplest visual identification [21] to densitometric and anatomical criteria of varying complexity [22,23,24,25,26,27,28]. However, when using them, errors and biases can occur [29,30,31] due to various reasons, ranging from the arbitrarinessof techniques and their interpretations to particular wood structure traits or anomalies, for example, intra-annual density fluctuations (IADFs).

The currently developing quantitative wood anatomy provides a more detailed insight into the processes of wood formation [7,32]. However, despite the successes already achieved, there is currently no single criterion that allows unequivocal assignment of cells to the earlywood, latewood, or transitional zone, even for conifers with their most “simple” wood structure [15,33]. In particular, the well-known Mork criterion [34] is an empirical value of the ratio of cell wall thickness tolumen set arbitrarily on a limited experimental basis, and the ambiguity of the interpretation of Mork’s original work led to the use of two different formulas [33]. Many authors have tried and are still trying to adapt the Mork criterion or derive their own [22,30,31,35,36,37,38]. This includes quantitative estimation of the tracheid form providing an optimal trade-off between water conductivity and mechanical strength [35], useof the radial-to-tangential cell diameter ratio as a criterion (ratio >1 for earlywood and <1 for latewood) [36], and modification of the Mork criterion value to particular site conditions and/or species based on comparison with visual, statistical, or densitometric criteria manually [30,31,37] or in specialized software [38].

We believe that the analysis of the statistical distribution of cells within the growth ring based on a large dataset of anatomical measurements can provide a functionally determined criterion for the distinction of zones that minimizes discrepancies and errors. This idea in its most basic form was created long time ago, using the weighted sum of two normal distributions for earlywood and latewood tracheids to describe their distribution by radial size for particular sites and species (see Figure 2.20 in [7]). However, it is still mostly untested, and the questions of its applicability and limitations remain open. In this study, we started answering these questions by testing the applicability of this new criterion to species with differing structural features that can make it difficult to apply existing approaches or lead to strong discrepancies between their results: a proportion of latewood in the ring, anatomical differences between earlywood and latewood tracheids, and abrupt or gradual transition [31,39,40,41]. Other factors leading to shifts or anomalies in the wood structure were kept to a minimum; thus, only mature wood for three conifer species from the same habitat from cold conditions (to exclude IADFs) was considered in this study. Therefore, the purpose of this work wasto create the basis for a universal model of the distribution of tracheids by zones, which can describe the anatomical structure of conifer rings, regardless of the species.

## 2. Results

### 2.1. Statistical Distributions of Tracheids in Terms of Radial Cell Diameter, Cell Wall Thickness, and Derivative Traits

**Figure 1:**
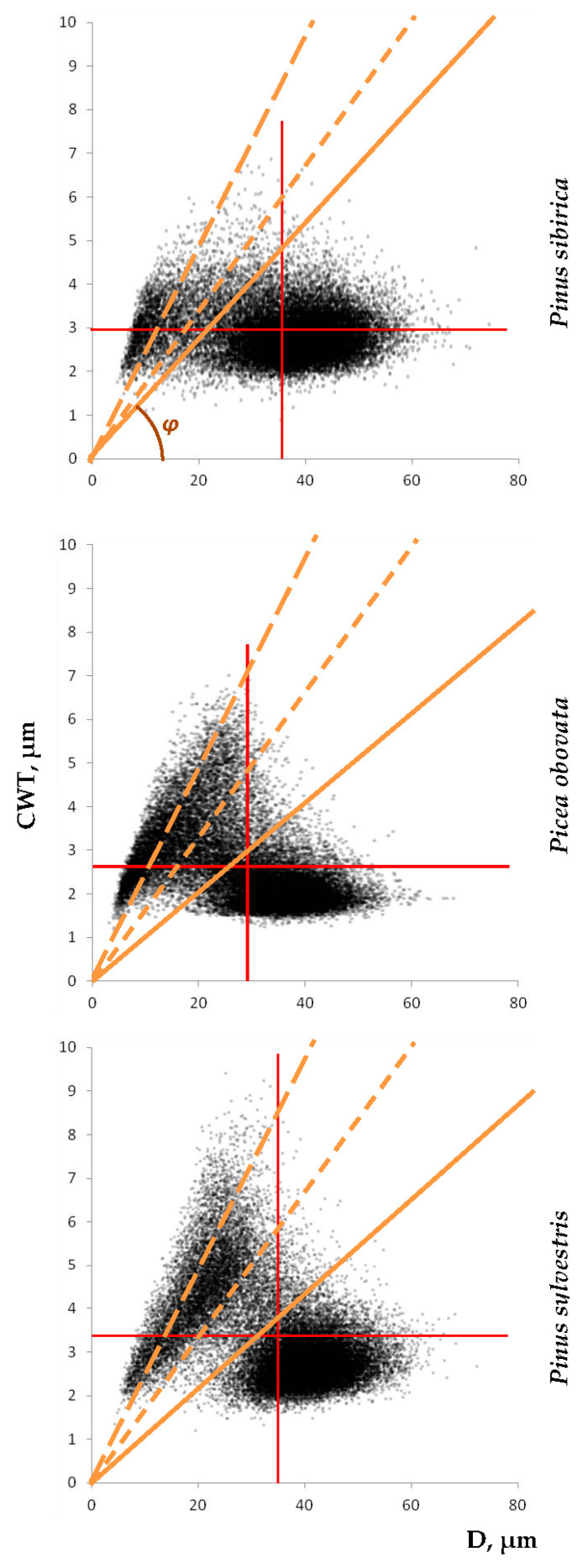
Scatter plots of the tracheid distribution in terms of radial size D and cell wall thickness CWT. Diagonal dashed lines represent the Mork criterion according to Equation (1) (CWT/D = 0.167; short dashes) and Equation (2) (CWT/D = 0.25; long dashes) by Denne [33]; diagonal solid line represents the species-specific mean CWT/D of transition wood calculated in this study. Vertical and horizontal lines mark the mean values of D and CWT. On the first panel, slope angle *φ* of CWT/D is presented.

The actual distribution of tracheids as laid out in the scatter plots of cell wall thickness (CWT) dependence on cell radial diameter (D) (Figure 1) shows the existence of two distinct dense clusters of cells in the earlywood (EW) and latewood (LW) areas, i.e., large thin-walled and small thick-walled cells, respectively, as well as a fairly large number of cells in-between these clusters, for all three species. These last cells can be assigned to the nearest cluster or allocated to the separate transition wood zone (TW) because they are usually spatially located between typical earlywood and latewood within the tree ring. It is obvious that the values of the Mork criterion CWT/D = 0.167 and 0.25 (according to both formulas given by Denne [33]) fall into the cluster of latewood cells or to the border of the transition zone closest to it for all three species. That is, for this sample of the experimental data, the Mork criterion in its original form is not accurate, and some lower value of CWT/D should be actually used.

**Figure 2:**
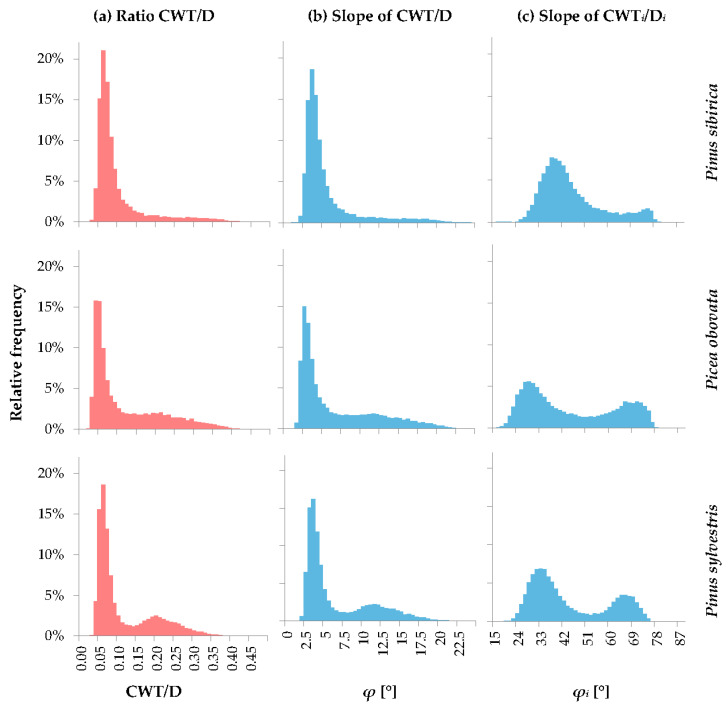
Probability density functions of the CWT/D ratio (**a**) and its slope *φ* before (**b**) and after (**c**) standardization of CWT and D. Values on the horizontal axis represent the lower boundaries of the corresponding bins, e.g., value 0.05 on the CWT/D axis corresponds to bin 0.05 ≤ CWT/D < 0.06. Bin width is 0.01 for CWT/D, 0.5° for *φ*, and 1.5° for *φ_i_*.

The next step is the change from the two-dimensional D–CWT plot to the one-dimensional variable, some value of which can be used as the criterion for distinction between tree-ring zones. The most obvious singular characteristic that can be used to investigate distribution of cells is the CWT/D ratio, i.e., the slope tangent of the direct proportionality line in Figure 1. For all considered species, it has a statistical distribution with a very pronounced and sharp maximum in the area of earlywood cells, but an indistinct and very elongated tail in the area of latewood, which makes it difficult to find a precise criterion for dividing the cells within the ring into zones (Figure 2a). The small proportion of latewood cells in the ring and the gradual transition from earlywood to latewood, which are characteristic of *Pinus sibirica*, make the latewood maximum of cell distribution even less pronounced for this species. The slope angle *φ* has distribution patterns similar to those of CWT/D (Figure 2b). However, the existence of clearly visible clusters in the CWT(D) scatter plots indicates the possibility of transformation that allows them to be separated.

The simplest such transformation is linear scaling, which can be performed by dividing both variables by their means. As a result of this scaling, the CWT and D values, as well as their ratio, transform linearly. Therefore, for the distribution of CWT/D, only the scale along the horizontal axis changes (not presented). However, the slope angle *φ* is described by a nonlinear albeit monotone arctangent function, so a change in the variability range of *φ* during linear scaling from 1.9–22.5° for raw measurements to 20–79° for indexes leads to a change not only in the scale of distribution functions but also in the shape of the *φ* values distribution. Indeed, the distributions of the slope angle for the indexes took on a distinctly bimodal shape for all three conifer species (Figure 2c).

### 2.2. Functional Modeling of the Statistical Distributions

**Figure 3:**
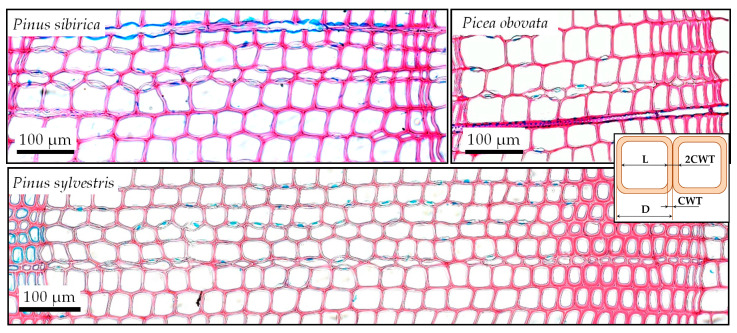
Examples of transverse wood sections of three conifer species in the study area. Insert contains scheme of measurements: initial row of subsequent lumen (L) and double cell wall thickness (2CWT) measured values, which are then automatically transformed into cell radial diameter (D = L + 2CWT) and cell wall thickness (CWT = 2CWT/2).

For *Pinus sylvestris*, the shape of the probability density curve is vaguely similar to the sum of two normal distributions obviously representing EW and LW. Forthe two other species, the presence of an additional component that does not fit into such a model is more noticeable, because alarge number of cells are located between two maxima. Taking into account the gradual nature of the anatomical structure transition observed in the considered conifers, i.e., the existence of cells intermediate in shape and location between earlywood and latewood (Figure 1 and Figure 3), this transition zone can be modeled with a generalized normal distribution with shape parameter *β* > 2. This form of statistical distribution can be described as the superposition of fluctuations due to random causes and normally distributed external factors, described by a normal distribution, on a uniform shift in the morphometric proportions of tracheids from typical EW to typical LW cells.

As for distribution components for EW and LW themselves, several unimodal forms were tested as possible distributions. Neither normal distribution nor wider-shaped bells of generalized normal distribution were able to describe these parts of the tree ring well enough to have chi-squared test significant at the level of 0.05 (not presented). To find a better solution, we took into account the obviously limited nature of the actual *φ_i_* values and the possibility of distribution skewness associated with intra-seasonal cell traits’ dynamics aside from separation into tree-ring zones (cf. nonconstant values of cell traits shown by average tracheidograms published in the previous study of these datasets [31]). Thus, we tested beta distribution as adhering to both the characteristics of limited range of variability and the possibility of skewness.

**Figure 4:**
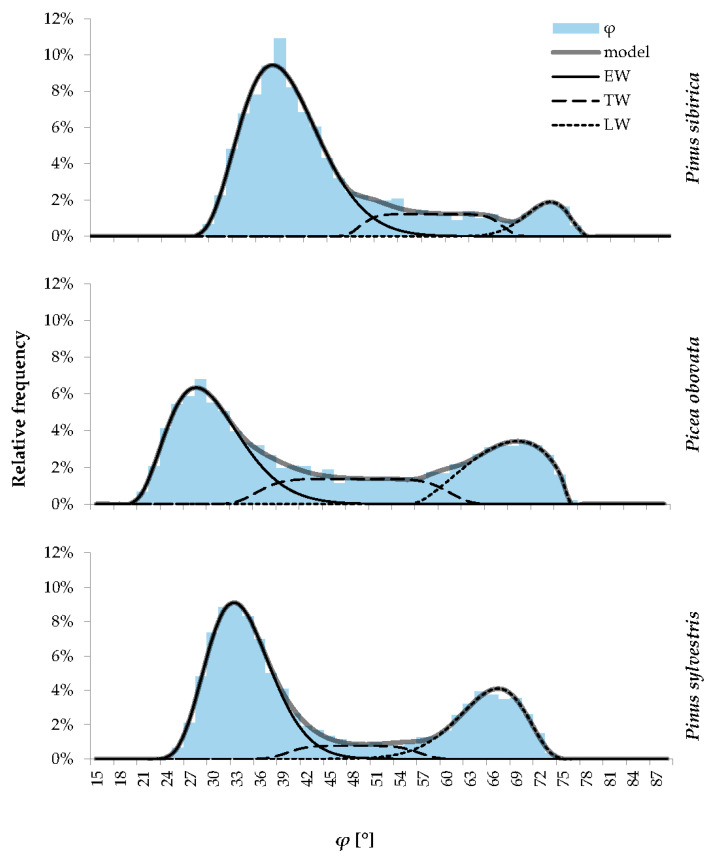
Actual (histogram) and modeled (thick solid line) probability density functions of the slope φ(CWT*_i_*/D*_i_*). Model (thick grey line) is a sum of beta distributions for earlywood (EW, solid line) and latewood (LW, dotted line) cells, and generalized normal distribution for transition wood (TW, dashed line). Values on horizontal axis represent the lower boundaries of the correspondingbins, as in Figure 2.

The process of fitting distribution parameters, carried out by the numerical least squares method on a mass material (thousands cells per species, Table 1) with a division of the variability range into bins of 1.5° width, made it possible to obtain distribution models that very accurately reflect the actual data (Figure 4) and successfully passed the chi-squared test for the total sample in *Pinus sylvestris* and *Picea obovata* rings, and for four out of five individual trees of *Pinus sibirica*. For one of the *Pinus sibirica* trees, the significance level was just below 0.05, and we could not find parameter values for the total *Pinus sibirica* dataset that allowed necessary significance level.

The obtained statistical characteristics of the model distributions in the cells of each zone of the ring are presented in Table 2. For *Pinus sylvestris* and *Picea obovata*, earlywood makes up 49–62% of the ring, and latewood is 29–30%, whereas for *Pinus sibirica*, earlywood is about three-quarters of the ring (72–81%), and the content of latewood cells is less than 9%. The transition wood zone is most pronounced in *Picea obovata* (22%) and least pronounced in *Pinus sylvestris* (8%). For all three species, the variability (standard deviation) of the slope angle is greatest for the transition zone due to the use of a generalized normal distribution with large value of scale parameter.

If one wants to split the ring into two zones without isolating the transition wood, we suggest using the center *μ* (mean value as well as the median and mode) of the symmetrical TW cell distribution as a threshold value, so that more EW-like half of the TW tracheids join the EW cluster, and the other half join the LW cluster. With the inverse transformation CWT/D = CWT_mean_/D_mean_·tg(*φ_i_*), we obtain threshold values *k* = CWT/D = 0.136 for *Pinus sibirica* (0.123–0.146 for individual trees), 0.105 for *Picea obovata*, and 0.110 for *Pinus sylvestris*, as shown by the solid diagonal line in Figure 1. With this division of the ring into two zones, we obtain an EW content of 84.5% (81.6–87.3%) for *Pinus sibirica*, 60.3% for *Picea obovata*, and 66.2% for *Pinus sylvestris*, whereas the rest of the ring (15.5, 39.7, and 33.8%, respectively) contains LW.

To divide the cells within a ring into three zones, one can, for example, use the values of *φ*_i_ at which the model distribution functions for neighboring zones (EW–TW and TW–LW) have equal values (the intersection points of the distribution curves in Figure 4) or the percentiles of the total distribution corresponding to the proportions of the corresponding zones in the ring. However, one should also take into account the irreversibility of the transition (especially for rings with fluctuations in wood density) and the fact that the first cells of the ring always belong to earlywood, even if due to lesser size; they can theoretically belong to TW by the numerical criterion.

In addition to the criterion for dividing the ring into two or three zones, the average values, modes, or medians of the ratio of cell morphometric parameters CWT/D in each zone of the ring, obtained from standardized data by inverse transformation, can be used as functional quantitative characteristics of the wood structure. For the considered samples, we obtained the estimates of the means for CWT/D = 0.068, 0.051, and 0.065 in earlywood and CWT/D = 0.332, 0.265, and 0.237 in latewood for *Pinus sibirica*, *Picea obovata*, and *Pinus sylvestris*, respectively.

## 3. Discussion

Previous studies of the *Pinus sylvestris* tracheids within the region showed that even for one species of conifer, the threshold value *k* = CWT/D adequately dividing the growth ring into earlywood and latewood varies from 0.09 to 0.16 depending on the environment [30,42]. We suggest that this may be due to the functional requirements of the mechanical strength of the xylem and the efficiency of water supply, which are determined both by the conditions of the habitat from the hot-dry forest-steppe to the cold-wet upper forest boundary, and by the prevailing height of the forest canopy and, accordingly, selected trees [12,43,44,45]. On the other hand, for the anatomical data of the three conifer species used in this study, but after averaging over five radial rows, a common threshold value of *k* = 0.125 was empirically fitted and used more or less successfully [31]. However, both from the scatter plots CWT(D) given in that previous study and from the plots for nonaveraged measurements in Figure 1, the actual boundary between the earlywood and latewood cells within a habitat is species-specific. There are significant differences between species in the shapes of scatter plots and in the location/density of earlywood and latewood clusters, reflecting the specific features of the anatomical structure. This variability reflects species-specific features with regards not only to tree size (height), but also to ecophysiological traits and species-specific adaptations to habitat conditions [46,47,48,49]. Taking into account the estimates of the threshold value *k* = 0.105–0.136 obtained in this work, we can conclude that the usage of one universal rigid threshold of *k*, as both interpretations of the Mork criterion suggest, quite often does not reflect the actual intra-seasonal variability of the anatomical structure in conifer wood.

Moreover, the necessity to take into account the species-specific traits of the wood structure was already noted, e.g., in the work of Denne [33], where the limits of applicability were illustrated by the examples of several species of the genus *Pinus*: *Pinus taeda*, for which a sharp EW/LW transition allows using both values, *Pinus pinaster* and *Pinus contorta*, for which latewood ratio varies greatly depending on the used *k* value due to a smooth, gradual transition, and finally *Pinus strobus*, for which both values of the Mork criterion fail to detect latewood at all (compare also with Carteni et al. [50]).

With regards to traits of particular species, it is also interesting that the less prominent LW zone in *Pinus sibirica* in terms of its proportion in the ring and anatomical distinctiveness is sometimes misinterpreted as lower wood strength. However, in this study, the CWT/D had higher mean values across all zones of the ring for this species than for its co-habitants, indicating less specialized functional wood properties with slightly higher mechanical strength throughout the ring. These particular anatomical traits make wood of stone pines and other such species, such asAmerican redwoods, mechanically strong but relatively lightweight, which apparently enables trees of those species to grow to quite impressive sizes [51,52].

In the proposed modeling approach, the standardization of anatomical measurements eliminates the shortcomings of previous attempts to empirically derive the value of *k*, such as severe asymmetry of distribution and indistinctive or absent bimodality. These issues result from features of the anatomical structure, such as a small proportion of latewood and weak variability in the morphometric parameters of tracheids along the ring, which are the scourge of, for example, white pines *Pinus cembra*, *Pinus sibirica*, and *Pinus strobus*. The proposed model adequately captures and describes the distribution of cells even in this case. A wider range of variability between individual trees, which can be seen in the low common signal observed in dendrochronological studies of this species (cf. [53] and references there), apparently is reflected in their anatomical features too, making inter-tree variations a source of noise in the local-scale model. However, the distribution of cells generally complies with the suggested model on the tree scale. This can be compared with age- and size-related trends in tree-ring width, which can wildly deviate from the exponential models in many trees for some species and conditions, but which can also conform for all or a certain subset of trees to one common regional curve (RCS approach) [54].

The inclusion of a separate distribution for transition wood in the model makes it possible to take into account and quantitatively describe characteristics such as the relative smoothness or abruptness of the transition from earlywood to latewood with great accuracy and quantitative description.

It should also be noted that the proposed model has another important advantage in contrast to existing approaches: the possibility of the theoretical justification and further use of the obtained quantitative estimates. For example, the means, modes or medians for the EW and LW zones can be interpreted as characteristics of the cross-section of tracheids that are optimal under given conditions and for a given species (or provenance, age strata, group of clones, and any other homogeneous group of trees) to perform the main xylem functions, water conductivity (EW), and mechanical strength (LW). The actual form of distribution of cells for these zones indicates the presence of actual optima in the actual traits of tracheids and fluctuations around them due, for example, to stochastic processes and climatic factors (the variability of which also by and large obeys the normal law). It should be noted that for all three species, the distributioncurves for earlywood and latewood practically do not intersect, confirming the need to add the transition zone in the model. The choice of generalized normal distribution is also theoretically justified by the very nature of the transition as a dynamic process rather than an instantaneous rearrangement of xylogenesis to the development of a functionally different xylem layer. This nature leads to a gradual shift in the anatomical characteristics of the tracheids in the transitional zone of the ring and introduces a similarity in its quantitative description with a uniform distribution against the background of the normal variability. The fact that the choice of these particular model equations of the statistical distribution made it possible to accurately capture the intricacies of the actual distribution curve, especially at the points of transition from zone to zone, confirms the plausibility of the above assumptions.

Among other things, the results confirm the stability of the obtained model and, accordingly, the stability of the functional anatomical structure of mature conifer wood in the conditions of the study area for 50 years; that is, in a wide range of current weather fluctuations, despite the possibility of light ring formation due to the early end of the warm season. Currently, it is still unknown whether this statement will be true for semiarid regions, where a lack of moisture can lead to IADFs of various localization and intensity.

Thus, we developed an approach that allows us to obtain a functionally substantiated anatomical criterion for dividing conifer rings into zones of earlywood, latewood, and, if desired, transition wood, taking into account the species and habitat. However, to assess the possibilities and limitations of its application, further research is needed in several directions, such as:

- The inclusion of the size–age dynamics of the anatomical structure in juvenile wood.
- The continuation of the analysis of the model stability when generalizing tracheidograms at different spatial scales.
- The analysis of model stability during years of growth depression and/or formation of anomalies in the anatomical structure (light rings, IADFs, etc.).
- The use of the obtained quantitative estimates of earlywood and latewood tracheids in the analysis of the influence of climatic factors on wood structure, etc.

## 4. Materials and Methods

### 4.1. Study Area and Sampling Site

This study was carried out on the Borus Ridge of the Western Sayan Mountains (South Siberia), in the vicinity of the Yenisei River. Most of the ridge area is covered with coniferous taiga forests (*Pinus sylvestris* L., *Pinus sibirica* Du Tour, *Larix sibirica* Ledeb., *Picea obovata* Ledeb., and *Abies sibirica* Ledeb.). Soils are loamy, thin, and stony with rock outcrops. The climate is sharply continental, with large daily and seasonal temperature fluctuations. Precipitation throughout the year is also unevenly distributed, with the maximum observed in July, the minimum in February–March. As the altitude increases, there is a decrease in temperatures and an increase in the annual amount of precipitation; therefore, the climate is cold and humid at the upper forest line, and the growth season is short and cool [55].

The sample collection site was selected on the eastern slope 50–100 m below the upper limit of tree distribution (52.81°N 91.51°E, 1300–1350 m a.s.l.; [31]), where several species of conifers grow under similar natural conditions.

Three species were chosen for this study: Scots pine (*Pinus sylvestris*), Siberian stone pine (*Pinus sibirica*), and Siberian spruce (*Picea obovata*). Of these species, *Pinus sibirica,* in contrast to the other two, has a very low latewood ratio and less pronounced differences in the anatomical structure between zones. On the other hand, the tree rings of *Pinus sylvestris* show a sharper transition between zones than the other two species (Figure 3). Thus, we could compare conifer wood with different latewood ratios, the contrast between earlywood and latewood, and the pattern of transition.

### 4.2. Sampling, Processing and Measurements

Wood samples (cores) were collected from living adult dominant and subdominant trees without mechanical damage, at least 15 trees of each species. The collection and transportation of cores were carried out using standard methods [56]. Cores were sampled from tree trunks at chest height (~1.3 m) perpendicular to the slope aspect with an increment borer with an inner diameter of 5.15 mm. They were packed into paper tubes for transportation. After drying, cores were mounted with PVA glue on wooden planks, and clean flat upper surfaces were obtained using a scalpel.

After measuring tree-ring width series for all cores on the LINTAB table (RINNTECH, Germany) with TSAPwin 4.68c software [57] and cross-dating them with COFECHA XP2007 software [58], 5 trees of each species older than 80 years (mostly 100–200 years old) were selected to exclude juvenile wood from anatomical measurements, considering that the last 50 rings closest to bark were used. The number of investigated trees is consistent with other wood anatomical studies (cf., for example, topic collection edited by Gennaretti et al. [59], where 2 to 9 trees were sampled for anatomical measurements in most of the studies).

Thin (~15–20 μm) transverse sections of wood were obtained from selected cores using a sliding microtome Microm HM 430 (Thermo Fisher Scientific, Waltham, MA, USA), stained with a mixture of 1% water solutions of the pigments safranin (red/purple, stains only lignified wood matter) and astra blue (stains indiscriminately), dehydrated with an increasing concentration of ethanol up to 96%, and fixed on glass slides in Canadian balsam. Microphotographs of these sections were taken using a biological microscope BX43 (Olympus, Tokyo, Japan) and a digital camera ProgRes Gryphax Subra (Jenoptik GmbH, Berlin, Germany) with a total magnification of 400×.

The anatomical measurements were performed over the last 50 years of growth (1965–2014 for *Picea obovata*, 1968–2017 for both *Pinus* sp.). For each ring, measurements were conducted in 5 radial rows of cells [60,61]. Their number (N), radial cell diameter (D), and cell wall thickness (CWT) were measured using the Lineyka 2.01 program [62]. For each ring (tree/year), measurements over radial rows of tracheids (tracheidograms) were normalized, i.e., stretched or compressed without distortion of cell trait values [63], to the mean cell number per row in the ring using the ProcessorKR 2.01 program [62] and then averaged to omit random differences between rows and highlight common intra-annual patterns in the tree-ring anatomical structure. The sample depth of the measurements is given in Table 1.

### 4.3. Derivative Criteria for Distinguishing between Tree-Ring Zones

In contrast to the CWT/L ratio (where L is the radial diameter of the cell lumen) used in two classical interpretations of the Mork criterion [33,34], which can reach indefinitely large values in latewood, the CWT/D ratio is strictly limited on both sides: CWT_min_/D_max_ ≤ CWT/D < ½. Here, CWT_min_ is the minimum cell wall thickness necessary to ensure the structural strength of the cell, which obviously exceeds the thickness of the primary wall of the cambial cell; D_max_ is the species-specific upper limitation of the cell radial size. The condition CWT/D < ½ follows naturally from geometric constraints considering L > 0. In addition, it should be noted that unlike lumen size, the morphometric parameters D and CWT can be tied to specific consequent stages of tracheid differentiation, i.e., radial cell growth by expansion and secondary cell wall deposition. That is, they are both carbon sinks and objects of growth processes’ regulation by internal and external factors [1,7].

In this case, we can use the equation L = D − 2CWT. Then, the original Mork criterion in the definition corresponding to Equation (1) by Denne [33], 4CWT > L, is expressed in terms of CWT/D as follows: 4CWT > (D − 2CWT), 6CWT > D, CWT/D > 1/6 ≈ 0.167. This is also the same value as the threshold suggested by Sviderskaya et al. [35]. Conversely, Equation (2) from Denne [33], 2CWT > L can be transformed as 2CWT > (D − 2CWT), 4CWT > D, CWT/D > ¼ = 0.25.

Similar to these criteria, if we use a certain threshold value CWT/D = *k* to distinguish between earlywood and latewood, this criterion will be displayed on the scatter plot CWT(D) as a straight line passing through the center of coordinates CWT = *k*·D, which can also be described through its slope angle *k* = tg(*φ*), *φ* = arctg(*k*) = arctg(CWT/D). Thus, for each measured cell, the values of *k* = CWT/D and the corresponding slope angles *φ* were calculated as variables for the possible tree-ring zone demarcation criteria. Their values are limited to the ranges 0 < *k* < 0.5 and 0 < *φ* < 90°.

For each tree species, the average values and range of variability (min–max) of cell parameters were estimated over the entire dataset of measurements (Table 1). Next, the morphometric parameters D and CWT were standardized (indexed), i.e., divided by the mean value, obtaining sets of indices D*_i_* and CWT*_i_*. Subsequently, the indexed CWT*_i_*/D*_i_* ratio and its slope angle *φ_i_*(CWT*_i_*/D*_i_*) were recalculated.

### 4.4. Modeling of the Cell Statistical Distributions

Distribution density functions for cells in the measurement datasets were modeled as the mixture distribution, i.e., the sum of several distributions, the weights (amplitudes *A*, %) of which belong to the convex combination, i.e., *A* > 0, and Σ*A* = 1.

For earlywood and latewood, to take into account possible skewness and limited nature of distribution, we proposed to use four-parameter beta distribution, for which the following parameters were estimated: shape parameters *α* and *β*, (both >1 to describe unimodal curve), the location parameters (full range of distribution) *a* and *b*. The formula for the distribution density of the generalized normal distribution was used as follows [64]:

(1) $$f\left(x\right)=\frac{1}{B\left(\alpha ,\beta \right)}\frac{{\left(x-a\right)}^{\alpha -1}{\left(b-x\right)}^{\beta -1}}{{\left(b-a\right)}^{\alpha +\beta -1}},$$

where $B\left(\alpha ,\beta \right)$ is the beta function $B\left(\alpha ,\beta \right)=\Gamma \left(\alpha \right)\Gamma \left(\beta \right)/\Gamma \left(\alpha +\beta \right)$, and Γ is the gamma function. For this distribution, the mean value is calculated as $\mu =\left(\alpha a+\beta b\right)/\left(\alpha +\beta \right)$; the mode (the most probable value) is $\mathrm{mode}=\left(\left(\alpha -1\right)b+\left(\beta -1\right)a\right)/\left(\alpha +\beta -2\right)$; the median (the point at which half the data are more and half the data are less than their value) is approximately $\mathrm{median}\approx \left(b-a\right)\left(\alpha -1/3\right)/\left(\alpha +\beta -2/3\right)+a$; and the standard deviation is $\sigma =\sqrt{\alpha \beta {\left(b-a\right)}^{2}/\left({\left(\alpha +\beta \right)}^{2}\left(\alpha +\beta +1\right)\right)}$.

For transition wood, which can be described as a distinctive zone of tree ring, where cell traits shift gradually from earlywood and latewood typical cell shape, generalized normal distribution was used. For this distribution, the mean value *μ*, the scale parameter *α*, and the shape parameter *β* were estimated. The formula for the distribution density of the generalized normal distribution was used as follows [65]:

(2) $$f\left(x\right)=\frac{\beta }{2\alpha \Gamma \left(1/\beta \right)}e^{-{\left(\left|x-\mu \right|/\alpha \right)}^{\beta }}.$$

If *β* = 2, such a distribution becomes normal, and for *β*→∞ tends to uniform distribution over the interval (*μ* − *α*, *μ* + *α*). For the generalized normal distribution, the mode and the median are equal to the mean value *μ*, and the standard deviation is calculated as $\sigma =\alpha \sqrt{\Gamma \left(3/\beta \right)/\Gamma \left(1/\beta \right)}$.

Amplitudes of distributions for earlywood and latewood cells in the overall mixture distribution were estimated, but the amplitude of transition wood distribution was calculated as the residual part of their sum in the model, Σ*A* = 1, i.e., *A*_TW_ = 1 − *A*_EW_ − *A*_LW_. Between two estimated amplitudes and 11 main statistical parameters (four for beta distributions and three for generalized normal distribution), the total number of parameters to be estimated was 13. The estimation was performed with iterative refinement using the maximum likelihood estimation (MLE) method in Microsoft Excel 2007.

The adherence of the model to actual data was tested in Microsoft Excel with Pearson’s chi-squared test (*χ*^2^) after division of the variability range into 50 bins of appropriate width and merging bins with less than 5 observations on the outskirts of the distribution. For the test, degrees of freedom were calculated as *m* − 13 − 1, where *m* is the final number of bins after merging, and 13 is the number of estimated distribution parameters. The model fits actual data adequately if the significance level of *χ*^2^ test is above 0.05.

## Figures and Tables

**Table 1:** Main characteristics of cell trait measurements: D, cell radial diameter; CWT, cell wall thickness; *φ* = arctg(CWT/D), slope of CWT/D ratio. Subscript *_i_* marks the indexed data.

Characteristics		Tree Species		
		*Pinus sibirica*	*Picea obovata*	*Pinus sylvestris*
Cover Period		1968–2017	1965–2014	1968–2017
Number of measurements	trees	5	5	5
	rings	249	245	249
	cells before/after averaging over 5 rows	24,565/4913	24,010/4802	31,450/6290
Cell traits: mean (min–max)	D, μm	35.3 (6.9–55.9)	28.6 (5.3–52.3)	34.5 (7.8–52.8)
	CWT, μm	2.9 (1.7–5.7)	2.6 (1.4–6.2)	3.3 (1.8–7.7)
	CWT/D	0.10 (0.04–0.41)	0.12 (0.03–0.39)	0.12 (0.04–0.36)
	*φ*, °	5.6 (2.5–22.5)	6.8 (1.9–21.5)	6.6 (2.4–20.0)
	D*_i_*	1.00 (0.20–1.58)	1.00 (0.18–1.83)	1.00 (0.23–1.53)
	CWT*_i_*	1.00 (0.60–1.98)	1.00 (0.55–2.36)	1.00 (0.55–2.32)
	CWT*_i_*/D*_i_*	1.20 (0.54–5.07)	1.33 (0.36–4.33)	1.21 (0.44–3.77)
	*φ_i_*, °	45.8 (28.4–78.8)	45.6 (20.0–77.0)	45.0 (23.8–75.2)

**Table 2:** Characteristics of the statistical distributions of tracheids by *φ_i_* = arctg(CWT*_i_*/D*_i_*), modeled for three tree-ring zones: earlywood (EW), transition wood (TW), and latewood (LW). Mean, median, and mode values of CWT/D for each zone were calculated from the same characteristics of *φ_i_*.

Zone	Parameter of Distribution ^1^	Tree Species		
		*Pinus sibirica* ^2^	*Picea obovata*	*Pinus sylvestris*
EW	*A*, %	77.4 (71.7–81.1)	49.2	62.0
	*μ*, °	40.52 (39.4–41.18)	30.57	34.60
	median, °	40.00 (39.64–41.81)	29.98	34.24
	mode, °	38.86 (38.33–39.79)	28.70	33.48
	*σ*, °	4.97 (3.22–5.68)	4.80	4.12
	CWT/D (mean/median/mode)	0.070 (0.065–0.075)	0.054	0.067
		0.069 (0.065–0.074)	0.053	0.066
		0.066 (0.063–0.070)	0.050	0.064
TW	*A*, %	14.2 (12.3–19.7)	22.1	8.4
	*μ*, °	59.00 (57.51–62.13)	49.00	48.81
	*σ*, °	5.26 (4.78–7.55)	7.35	5.13
	CWT/D (mean = median = mode)	0.136 (0.123–0.146)	0.105	0.110
LW	*A*, %	8.4 (6.6–8.6)	28.7	29.6
	*μ*, °	73.13 (72.02–74.22)	68.67	65.86
	median, °	73.42 (72.64–74.45)	69.01	66.37
	mode, °	74.11 (73.94–75.10)	70.29	67.48
	*σ*, °	2.61 (1.72–2.92)	4.38	4.44
	CWT/D (mean/median/mode)	0.270 (0.264–0.293)	0.233	0.216
		0.275 (0.268–0.298)	0.237	0.221
		0.287 (0.278–0.314)	0.254	0.233
*χ* ^2^		40.23 (15.47–27.82)	22.60	28.81
degrees of freedom		18 (16–17)	23	20
significance level		0.002 (0.047–0.49)	0.48	0.09

^1^ *A*, proportion of zone in the tree ring; *μ*, mean value of *φ*; *σ*, mean quadratic (standard) deviation of *φ*; *χ*^2^, chi-squared test value. ^2^ Values in brackets are the minimum and maximum values for models fitted to datasets of individual trees.

## Data Availability

The data presented in this study are available on reasonable request from the corresponding author.
